# Opposite rheological properties of neuronal microcompartments predict axonal vulnerability in brain injury

**DOI:** 10.1038/srep09475

**Published:** 2015-03-30

**Authors:** Thomas Grevesse, Borna E. Dabiri, Kevin Kit Parker, Sylvain Gabriele

**Affiliations:** 1 Mechanobiology & Soft Matter Group, Interfaces and Complex Fluids Laboratory, Research Institute for Biosciences, CIRMAP, University of Mons, 20 Place du Parc B-7000 Mons, Belgium; 2 Disease Biophysics Group, Wyss Institute for Biologically Inspired Engineering, School of Engineering and Applied Sciences, Harvard University, 29 Oxford Street, Cambridge, MA 02138, USA

## Abstract

Although pathological changes in axonal morphology have emerged as important features of traumatic brain injury (TBI), the mechanical vulnerability of the axonal microcompartment relative to the cell body is not well understood. We hypothesized that soma and neurite microcompartments exhibit distinct mechanical behaviors, rendering axons more sensitive to a mechanical injury. In order to test this assumption, we combined protein micropatterns with magnetic tweezer rheology to probe the viscoelastic properties of neuronal microcompartments. Creep experiments revealed two opposite rheological behaviors within cortical neurons: the cell body was soft and characterized by a solid-like response, whereas the neurite compartment was stiffer and viscous-like. By using pharmacological agents, we demonstrated that the nucleus is responsible for the solid-like behavior and the stress-stiffening response of the soma, whereas neurofilaments have a predominant contribution in the viscous behavior of the neurite. Furthermore, we found that the neurite is a mechanosensitive compartment that becomes softer and adopts a pronounced viscous state on soft matrices. Together, these findings highlight the importance of the regionalization of mechanical and rigidity-sensing properties within neuron microcompartments in the preferential damage of axons during traumatic brain injury and into potential mechanisms of axonal outgrowth after injury.

Microcompartments are an essential design feature in mammalian cells. For instance, motile cells use filopodia and lamellipodia to probe their mechanochemical environment and to orient their movement^1^,^2^, while cilia at the tip of ciliated cells are essential for sweeping the mucus and foreign particles out of the lung and trachea^3^. Compartmentalization is also prominent in neuronal function: neurons possess cable-like microcompartments (dendrites and axons) that propagate information in the form of action potentials, whereas the neuronal body microcompartment (soma) houses most of the genetic content and is the site of a large part of the protein synthesis. This compartmentalization is especially relevant in understanding the cellular manifestations of traumatic brain injury (TBI). Currently, it is proposed that the initial event in TBI is the pathological strain of axons as the result of an inertial loading^4^. This mechanical deformation is thought to damage the internal structure of axons causing diffuse axonal injury (DAI), which is one of the most common and important pathological features of TBI^5^,^6^.

To date, a unifying model of axonal degeneration considers that nerve insults lead to impaired expression of a local axonal survival factor, which results in increased intra-axonal calcium levels and calcium-dependent cytoskeletal breakdown^7^. Damage to neurofilaments and microtubules typical of axonal focal swellings can arise from stress-induced cell membrane poration, leading to Ca^2+^ ion entry and subsequent activation of calpains that degrade proteins non-specifically^8^. Alternatively, integrins, which are transmembrane proteins that physically couple the neuronal cytoskeleton to the extracellular matrix^9^ (ECM), have been shown to be an important contributor to DAI by propagating mechanical forces through the cytoskeleton^10^. In contrast, the soma is seemingly unaffected by mechanical insult. Although several reports have indicated shrunken somas^11^ with pycnotic nuclei (i.e. condensation of chromatin leading to a shrunken nucleus) or DNA damage^12^ after brain injury, important differences in the rate of degeneration between soma and cell processes must be taken into account. Indeed, prominent axonal pathology often precedes cell body loss that arises from the gradual degeneration of axons toward the cell body. Central to understanding the induction of axonal pathology is deciphering the mechanical vulnerability of the axonal microcompartment over the cellular body.

We hypothesized that specific cytoskeletal organization within neuronal microcompartments may lead to distinct rheological properties that potentiate a greater vulnerability of axons to injury. To test this, we combined micropatterning with magnetic tweezers to apply local stresses to individual microcompartments of bipolar neurons. We found that the rheological behaviors of soma and neurite were dominated by elastic and viscous properties, respectively. Mechanical testing of neuronal microcompartments treated with pharmacological agents causing specific cytoskeletal disruption further indicated that neurofilaments and microtubules were the principal mechanical load bearing elements of the neurite, whereas the rheology of the soma was dominated by the nucleus. Furthermore, we assessed whether the rheological properties of both neuronal microcompartments can be affected by stiffness changes of their microenvironment, as observed in many injury-related pathological responses. We found that the neurite compartment tuned its internal stiffness to match the compliance of the substrate and adopted a pronounced viscous state on soft microenvironments. In contrast, the cell body was insensitive to matrix stiffness changes and remained characterized by a solid-like behavior. Taken together, our findings suggest that the preferential damage of axon over other neuronal microcompartments in brain injury is related to opposite rheological properties in neuronal microcompartments that lead to a greater vulnerability of the neurites, as observed in DAI.

## Results

### Combining protein micropatterns and magnetic tweezers to probe the rheological properties of neuronal microcompartments

**Figure 1:**
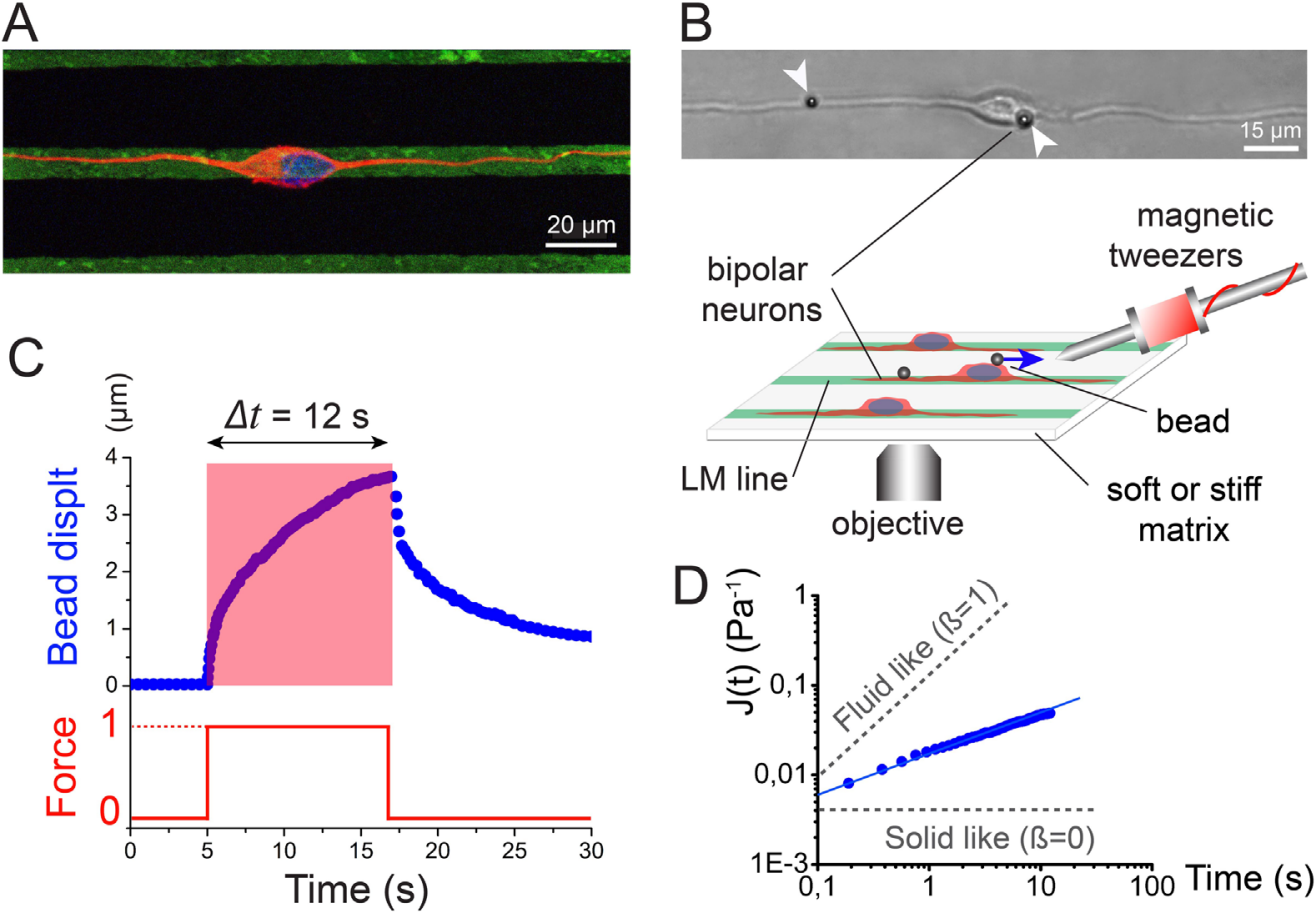
Rheological characterization of cortical neuronal microcompartments. (A) Immunofluorescence image of microtubules (red) and nucleus (blue) of a bipolar neuron adhering to 10 μm wide laminin lines (green). The scale bar is 20 μm. (B) Schematic description of the experimental setup for probing rheological properties of neuronal microcompartments with magnetic tweezers. Individual cortical neurons were grown on LM stripes (green) to impose a reproducible bipolar morphology. The inset DIC image depicts a bipolar neuron with magnetic beads attached to the neurite and the soma (white arrowheads). The scale bar is 15 μm. (C) Temporal evolution of a typical bead displacement curve (blue data) in response to a single force protocol (red line). (D) Logarithmic representation of the creep function *J(t)* obtained from the bead displacement *d(t)*. Gray dashed lines represent solid-like (*β* = 0) and fluid-like (*β* = 1) behaviors.

We proposed that differences in the mechanical properties of individual neuronal microcompartments (neurite and soma) potentiate the greater vulnerability of neurite towards a mechanical insult. To test this, we measured the creep response of individual neuronal microcompartments by applying a constant force pulse with magnetic tweezers and following the resulting deformation. It is well established that studying deformation under constant stress (creep experiment) can reveal key viscoelastic parameters of single living cells^13^,^14^. To facilitate access to individual neuronal microcompartments and to ensure the reproducibility of our rheological tests, we plated single cortical neurons on lines of laminin (LM) of 10 μm wide obtained by microcontact printing. As shown in Fig. 1A, LM micropatterns guided neurite extension and imposed a reproducible bipolar morphology to individual cortical neurons. Magnetic forces were applied in the *x,y* plane to obtain local stretching deformations of both neuronal microcompartments. The creep function was derived by following the bead displacement, *d(t)*, (Fig. 1C) for individual neuronal microcompartments (Fig. 1B inset, Supplementary Movies S1 and S2) when deformed with a constant force, *f_0_*, and taking into account the specific geometries of both neuronal microcompartments (Supplementary Fig. S1). The creep function *J(t)* (Fig. 1D, expressed in units of Pa^−1^) was then calculated using the ratio between the strain *ε(t)* and the stress *σ_0_*^15^, such as:
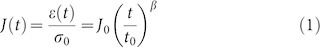


We found that the creep response of both neuronal microcompartments could be fitted with a time power law (Eq. 1, Fig. 1D), which has been used previously to describe the mechanical properties of many different cell types, with different techniques and over a large range of timescales and frequencies^16^,^17^. The parameters *β* and *J_0_* of the power law can be used as metrics to compare the stiffness and the viscoelastic properties of different materials, respectively. The power law exponent *β* characterizes the time-dependent viscoelastic properties, where *β* = 0 corresponds to an elastic solid and *β* = 1 to a viscous fluid (Fig. 1D). At intermediate values of *β*, both elastic and dissipative mechanisms coexist. The inverse of the prefactor, 1/*J_0_*, is equivalent to a shear modulus at time *t* = 1 s. We sought to compare the mechanical differences between neuronal microcompartments using the power law parameters *β* and *J_0_*. For clarity and comparison with other studies, the stiffness of both microcompartments will be expressed using Young's modulus (see Supplementary Information for further details).

**Figure 2:**
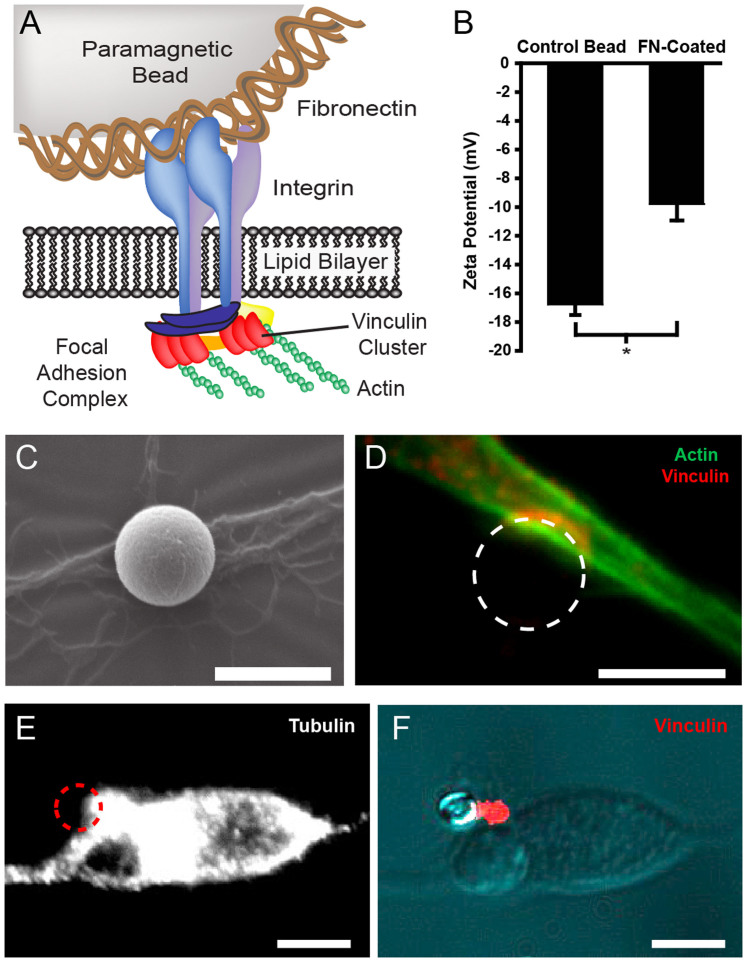
FN-coated paramagnetic beads bound specifically to neurons. (A) Schematic of the interaction between a superparamagnetic bead, fibronectin and the neuronal cytoskeleton through the recruitment of transmembrane integrins to form a focal adhesion complex. (B) The magnetic bead coating was confirmed by measuring the zeta potential of uncoated beads (control) and FN-coated beads with n = 7 bead reactions per condition. (C) SEM image depicting a FN-coated paramagnetic bead bound to a neurite after beads were seeded onto neurons for 20 minutes. Scale bar = 5 μm. (D) Immunostaining for vinculin (red) and actin (green) revealed focal adhesions at site of bead binding. Scale bar = 5 μm (E) Immunostained image of tubulin in the cell body with a bounded magnetic bead depicted by a red dashed circle. Scale bars = 10 μm. (F) The FN-coated bead is linked to the cytoskeleton through the formation of vinculin-containing adhesion sites that colocalize with the microtubule network. Scale bars = 10 μm.

Magnetic forces were applied on 4.5 μm diameter superparamagnetic beads, which maintain magnetism only in the presence of an external magnetic field while also having a high magnetic susceptibility. We used epoxy-activated paramagnetic beads that were pre-activated, uniform and silica-based beads coated with high-density epoxy functional groups on the surface (see the method section for further details). Epoxy-activated beads bind nucleophiles such as hydroxyl groups within proteins, providing an extremely stable linkage between the ligand and the matrix. Superparamagnetic beads were coated with fibronectin (FN) to bind specifically to the cytoskeleton of neurons via integrins (Fig. 2A). Indeed, focal adhesion complexes formed between the bead and the neuron at the cytoplasmic tail of clustering integrins. Focal adhesions are composed of several proteins responsible for mechano-chemical signalling that serve to transduce extracellular mechanical signals to the neuronal cytoskeleton^18^,^19^. This relationship creates a mechanical continuity between the bead and the internal scaffolding of the neuron. Before adding beads to neurons, zeta potential analysis was used to confirm that beads were coated with FN. Relative to the negatively charged epoxy-coated surface of uncoated beads, FN-coated beads exhibited less negative zeta potentials (Fig. 2B), indicating that FN was bound to the bead surface, reducing the net negative charge^20^.

Since the height of the cell body of neurons was about 8 μm and the diameter of neurites about 1.5 μm, we selected an intermediate diameter of magnetic beads of 4.5 μm, which created a sufficient adhesive interface with both neuronal microcompartments while preventing the complete bead internalization. FN-coated beads bound to the apical surface of neurons (Figs. 1B and 2C) and formed specific links with the intracellular cytoskeleton (Figs. 2C and 2E) through the formation of vinculin containing adhesion sites (Figs. 2D and 2F). Subsequently, neurons and beads were gently rinsed in a warm buffer solution to wash away any unbound beads and magnetic tweezer experiments were performed for a maximum of 40 minutes to maintain a low degree of bead internalization in both neuronal microcompartments (Figs. 1B, 2C and 2F). Together, these experimental precautions permitted to form specific links between FN-coated beads and the neuronal cytoskeleton, while minimising bead internalization.

### Neuronal microcompartments display opposite rheological behaviors

**Figure 3:**
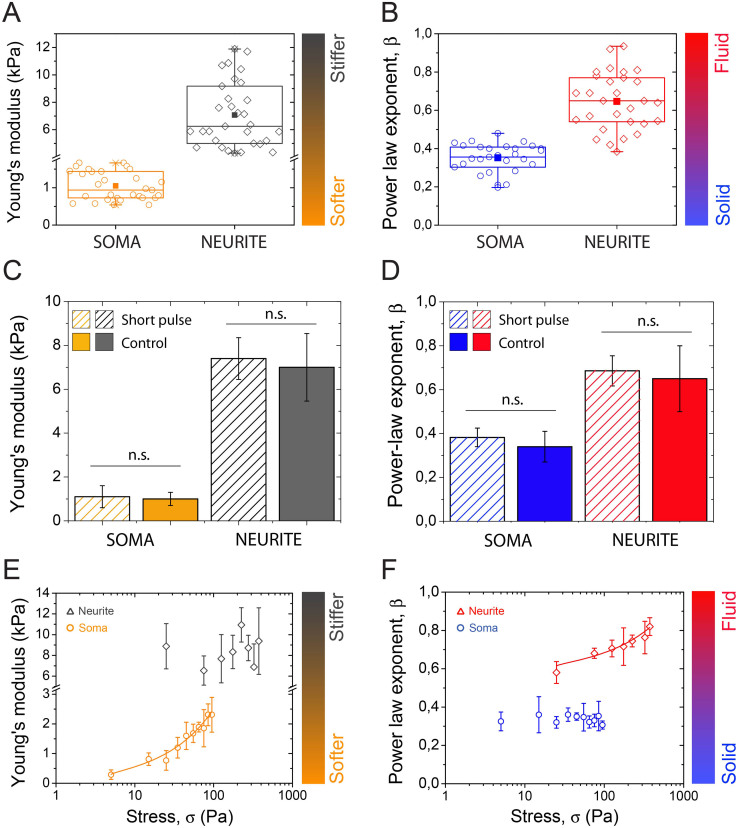
Comparison of the mechanical behavior of neuron microcompartments: soma vs neurite. (A) Young's modulus, obtained from 1/*J_0_*, for the soma (*n* = 31, in orange) and the neurite (*n* = 30, in dark gray) compartments. (B) Power-law exponent *β* for the soma (*n* = 31, in blue) and the neurite (*n* = 30, in red) compartments. Time dependence of (C) the Young's modulus and (D) the power-law exponent *β* for the soma and the neurite microcompartments. Creep experiments were performed with short (Δt = 850 ms, dashed data) and long (Δt = 12 s, plain data) force pulses on both neuron microcompartments. Force dependence of (E) the Young's modulus and (F) the power law exponent β for the soma (circles) and the neurite (lozenges) microcompartments. Data are mean ± SD, with *n* = 14 for short pulses, *n* = 31 for long pulses and 5 ≤ *n* ≤ 7 for (C) and (D).

We determined that the soma was the softest neuronal microcompartment with a mean Young's modulus of 1.05 ± 0.38 kPa (Fig. 3A, *n* = 31), whereas the neurite was more than 6 times stiffer with a mean Young's modulus of 7.0 ± 2.4 kPa (Fig. 3A, *n* = 30), which is close to the value of 12 kPa reported by Bernal and coworkers for PC-12 neurites^21^. In addition, the soma was characterized by a mean power law exponent *β* = 0.34 ± 0.07 (Fig. 3B, *n* = 31), which was two times lower than the *β* value observed for the neurite *β* = 0.65 ± 0.15, *n* = 30). These power law exponents indicate that the soma behaved like an elastic solid, whereas the neurite was significantly more viscous-like. To the best of our knowledge, such a large exponent has not been reported before in mammalian cells, which are typically characterized by *β* exponents ranging from 0.15 to 0.5^15^. The *β* exponent value that we observed for the neurite corresponds to the limit predicted for semiflexible polymers^22^. In the context of brain injury, our results suggest that a mechanical insult will impact neuronal microcompartments differently. Indeed, the viscous-like nature of neurite will allow the dissipation of most of the mechanical stress, in comparison to elastic-like behavior of the soma that will tend to store a large amount of the energy. The stress may dissipate through damage to the internal neurite structure, providing an explanation for the greater vulnerability of neurite in TBI.

Considering that the mechanical properties of cells are generally dependent on time-scales and the magnitude of stress, we sought to extend our mechanical characterization of neuronal microcompartments to shorter time-scales and varying stresses that can recapitulate brain injury conditions. To this end, we first abruptly deformed neurons at constant stress, *σ_0_*, with short magnetic pulses (Δt = 850 ms). We found that the Young's modulus (Fig. 3C) and the power law exponent *β* (Fig. 3D) of both microcompartments were statistically similar for short (Δt = 850 ms) and long (Δt = 12 s) force pulses. These results demonstrate that the mechanical behaviors of somas and neurites are time-scale independent, within the range probed.

We then measured the force-dependence of the creep parameters *β* and *J_0_* to further characterize the behavior of both neuronal microcompartments in response to higher mechanical loads. Previous reports have shown that eukaryotic cells can exhibit non-linear behaviors such as “stress stiffening” (increase of 1/*J_0_* with force), which may be ascribed to cells with small internal prestress, or “fluidization” (increase of *β* with force), which is related to stiffer and more elastic cells^15^. We probed somas with stresses ranging from 5 to 100 Pa and neurites with stresses ranging from 20 to 400 Pa to assess the evolution of power law parameters with stress. These experiments indicated that the cell body followed a stress-stiffening behavior, as demonstrated by the increase of the Young's modulus with stress (Fig. 3E), while it remained solid-like with no significant changes in the *β* parameter (Fig. 3F). In contrast, the neurite compartment fluidized significantly (increase of *β* exponent) with the applied stresses in the range 20–400 Pa (Fig. 3F), and failed to show stress-stiffening behavior (Fig. 3E). It is interesting to note that the stress-stiffening behavior was not dependent on the type of mechanical stimulation. Indeed, we observed the same evolution of the Young's modulus (obtained from 1/J_0_) of the soma with stresses applied from compressing or stretching deformations (Supplementary Fig. S2).

Taken together, these results demonstrate that cortical neurons possess microcompartments with opposite viscoelastic behaviors: the soma is soft and behaves as an elastic solid, whereas the neurite is significantly stiffer and viscous-like. Force-dependent experiments have shown that the rheological differences between both microcompartments are enhanced in response to larger forces with a stress-stiffening of the soma and a fluidization of the neurite. Collectively, these data ask a fundamental question about the role of the cytoskeleton in the establishment of the disparate mechanical response of neuronal microcompartments.

### The nucleus is responsible for the viscoelastic properties of the soma

The cytoskeleton has been shown to be responsible for the mechanical behavior of many cells types^23^,^24^. Cytoskeletal filaments in the axons are disrupted in DAI^25^,^26^, leading to the typical axonal swelling. We sought to determine which components of the cytoskeleton were the load bearing elements of neuronal microcompartments and therefore more prone to damage during TBI. In order to address the role of cytoskeleton in the mechanical behavior of neuronal microcompartments, we measured the creep function of neuronal microcompartments treated with different drugs that selectively depolymerize various types of cytoskeletal filaments or inhibit the action of their associated molecular motors. Power law rheology has been shown to be a universal property of adherent cells and previous reports have demonstrated that *J_0_* and *β* remain valid parameters, even when cells were treated with an exhaustive range of cytoskeletally active drugs^27^,^15^.

**Figure 4:**
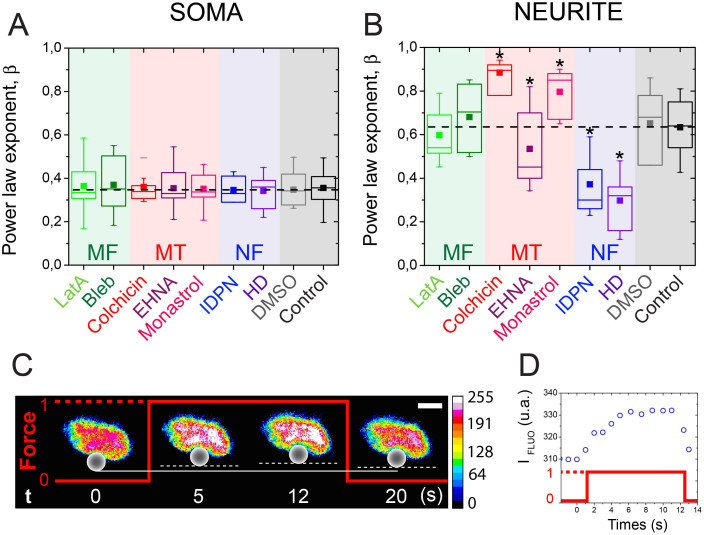
Rheological role of individual cytoskeletal components. Effect of the inhibition of cytoskeletal components on the power law exponent *β* of (A) cell body and (B) neurite microcompartments of cortical neurons. Dashed black lines show the mean β value obtained for normal microcompartments (12 ≤ *n* ≤ 14 for drug-treated cells and *n* = 31 for normal cells). Asterisks indicate significant changes with control (p < 0.01). (C) Successive changes of the level of chromatin condensation in response to a constant force applied to the soma. Intensities of DNA staining were digitized in 256 bits and color-coded (from high to low: white, purple, red, orange, yellow, green, light blue and dark blue). Highly condensed domains are represented by higher fluorescence intensity with respect to the less condensed ones. (D) The normalized intensity of the nucleus stained with Hoechst increases during the creeping phase.

The soma of neurons comprises the nucleus, cytoplasmic organelles and three types of fibrillar elements: microtubules (MTs), neurofilaments (NFs) and actin microfilaments (MFs). We used different pharmacological agents to disrupt each class of cytoskeletal filaments and to inhibit the action of their associated molecular motors (see Supplementary Information for further details). The concentrations of the pharmacological agents were chosen to significantly disable MTs^28^, NFs^29^ and MFs^30^ (see the method section for details), as verified by fluorescent microscopy. The specific alteration of MTs, NFs and MFs and their associated molecular motors (kinesin and dynein for MTs, myosin for MFs) did not significantly change the power law exponent value of the soma (Fig. 4A). In contrary to the neurite microcompartment (Fig. 4B), our results suggest that the viscoelastic state of the soma is a robust parameter that does not depend on the cytoskeletal network. Based on this intriguing observation, we further characterized the internal organization of neuronal cell bodies by laser-scanning confocal microscopy. We observed that the soma of cortical neurons contained a prominent nucleus that occupied most of its internal volume (Supplementary Fig. S3). Free-spaces of the cell body were filled with MTs that form a thin net surrounding the nucleus (Supplementary Movie S3), whereas MFs were concentrated at both extremities of the soma.

These observations suggest that the nucleus plays a significant role in the viscoelastic response of the soma. In support of this hypothesis, it is interesting to emphasize that the *β* exponent observed for the soma (*β* = 0.34 ± 0.07) is very close to the power law exponent of 0.32 reported by Dahl and colleagues for the nuclei of epithelial cells^31^. The selective disruption of intermediate filaments was characterized by an important decrease of the Young's modulus of the soma (*E* = 0.70 ± 0.13 kPa, Supplementary Fig. S4A), suggesting that nuclear structural components such as lamins, which are type V intermediate filament proteins, are involved in the mechanical rigidity of the cell body.

To further confirm the role of the nucleus as the prominent aspect of the soma mechanics, we measured nuclear deformation during a typical creep experiment with fluorescence microscopy. We used the Hoechst fluorochrome that intercalates between successive DNA base pairs in order to relate variations of the fluorescence intensity to changes in the condensation state of chromatin^32^. At zero force conditions, the Hoechst intensity level was homogeneous within the nucleus and about three times more elevated than the background (Fig. 4C). During the application of a constant force pulse via a paramagnetic bead bound to the cell body, we observed an important elevation of the fluorescence intensity in the projected nuclear area (Fig. 4D and Supplementary Movie S4). The nuclear zones of high fluorescence levels, which correspond to approximately 29% of the nuclear area (Fig. 4C, *t* = 12 s), are related to the formation of highly condensed chromatin domains that arise from local cell body deformation.

### Sliding neurofilaments contribute to the neurite fluidization

The cytoskeleton of neurites is composed of longitudinally aligned MTs and NFs along its long axis with a ratio of NF/MT of about 10:1^33^ and axons are characterized by ringlike structures of MFs that are wrapped around their circumference with a periodicity of 180 to 190 nanometers^34^. We performed creep experiments on drug-treated neurites to investigate the individual role of MFs, NFs, MTs and their associated molecular motors (myosin II for MFs, cytoplasmic dynein and kinesin-5 for MTs) in the viscoelastic response of neurites submitted to a radial deformation. Disrupting actin MFs (*β* = 0.60 ± 0.13) and inhibiting myosin II (*β* = 0.68 ± 0.16) did not significantly change the viscoelastic state of the neurite (Fig. 4B), suggesting that actomyosin contractility does not play a major role in the mechanical state of this compartment. Interestingly, this hypothesis is supported by the low level of MFs in neurite, which are represented by short cortical actin filaments organized into ring-like structures^34^ and thin protrusions perpendicular to the cell axis. The depolymerization of MTs increased the neurite *β* exponent to 0.88 ± 0.07, indicating that the neurite compartment becomes significantly more viscous when MTs are depolymerized. In addition, we observed that inhibiting the ATPase activity of the cytoplasmic dynein with EHNA decreased the *β* exponent significantly, whereas inhibiting the ATPase activity of the kinesin 5 with monastrol led to a higher value of the *β* exponent (Fig. 4B). Taken together, these data indicate that the antagonistic activities of microtubule motor proteins dynein and kinesin 5 are involved in the rheological properties of neurites, supporting the role of MTs in the fluidization of the neurite compartment. In contrast, disorganizing NFs decreased the *β* exponent of neurites to 0.37 ± 0.13 (Fig. 4B), which corresponds to a more pronounced elastic state.

Overall, our findings demonstrated that elastic and viscous components of the neurite viscoelastic response were MTs and NFs, respectively. The elastic behavior attributed to MTs was consistent with their large persistence length (lp ~ 5.2 mm), corresponding to a high bending modulus (*E_B_* ~ 2.15 10^−23^ Nm^2^)^35^ that allows MTs to act as rigid rods that resist against deformation. In contrast, NFs are very flexible polymers with a weak persistent length (lp ~ 150 nm) that prevents them from exerting an elastic role. Remarkably, this mechanical interpretation of the individual role of MTs and NFs in the establishment of the viscous-like behavior of a neurite is supported by its cytoskeletal composition. Indeed, NFs are the most abundant fibrillar components of the axon^36^,^37^ and were estimated to be on average 5–10 times more common than MTs, depending on the axonal diameter. The predominant contribution of NFs in the viscous behavior correlates with the worm like chain (WLC) theory for semi flexible polymers, suggesting that unbinding of weak attractive bonds between polymer filaments is the dominating mechanism of energy dissipation^15^. One may therefore speculate that the fluid-like behavior of MT-disrupted neurites is related to the large flexibility of NFs, whereas the fluidization of the neurite in response to large force values may arise from sliding friction between NFs through the breaking of cross-linking bonds^38^.

To examine this, we measured the creep response of neurite treated with 2,5-hexanedione (HD) that cross-links NFs. We observed that HD treatment increased the neurite Young's modulus (Supplementary Fig. S4B) as well as its elastic behavior, which was characterized by a low *β* exponent (Fig. 4B). Our results are in agreement with the WLC theory that predicts that increasing the cross-linking density will increase the Young's modulus and the elasticity of the polymer network. Based on these results, we suggest that neurofilaments are the most important load-bearing elements of the neurite and that their disorganization is at the onset of TBI-induced neuropathology.

To present a more complete picture of the behavior of neuronal microcompartments during injury events, one must also consider their individual response to the modification of their mechanical microenvironment. Indeed, several reports demonstrated that neurons can sense their mechanical environment and change their physiological properties accordingly^39^,^40^,^41^,^42^. In addition, recent works have shown that local tissue stiffness is likely to change during injury-related pathological processes, such as glial scar^43^, intracranial pressure^44^ or local inflammation^45^. To address this issue, we probed the rheological properties of both neuronal microcompartments in response to the modification of matrix stiffness.

### Adaption to the matrix stiffness is restricted to the neurite microcompartment

The effect of the substrate stiffness on the rheological properties of neuronal microcompartments was determined by performing creep experiments on cortical neurons cultured on soft hydrogels of 3.5 kPa, which was more than two orders of magnitude lower than the rigidity (*E* = 500 kPa) used in the previous sections. Soft hydrogels were microprinted with LM lines of 10 μm width in order to impose a bipolar morphology to primary cortical neurons (Supplementary Fig. S5). The adhesive ligand density on soft and stiff matrices was consistently controlled by fluorescence microscopy to avoid any influence of the protein coating^46^. In addition, fluorescent beads were embedded in soft matrices to control that magnetic pulses directed on soma and neurite microcompartments did not lead to substrate deformations, which may affect rheological measurements.

After four days in culture on soft polyacrylamide (PA) gels, cortical neurons extended neurites along the 10 μm wide LM lines to adopt a well-defined bipolar morphology and formed mature focal adhesion sites (Supplementary Fig. S6). By using vinculin as a marker of focal adhesions (FAs), we have quantified the global amount of FAs on soft (Fig. S6A) and stiff (Fig. S6B) substrates. Our findings indicated that bipolar neurons formed statistically the same amount of FA sites per cell on soft or stiff matrices (Supplementary Fig. S6C). Furthermore, the individual analysis of each neuronal microcompartment revealed a uniform distribution of FAs on soft and stiff matrices (Supplementary Fig. S6D). Together, our results suggest that cell-substrate adhesions remained constant on different matrix rigidities and thus may not affect the measured bead displacements between soft and stiff matrices.

Furthermore, recent works suggest that adherent cells may adapt their response to the nature of the matrix^47^. Although the influence of the chemical nature of compliant matrices on cell fate is still under debate in the literature^48^, we investigated whether the nature of PA matrices may lead to rheological modifications of neurons. To do this, we cultured cortical neurons on stiff microprinted PA matrices (E ~ 425 kPa). Very stiff PA hydrogels were prepared by increasing the polyacrylamide monomer concentration to 30% at a fixed monomer/crosslink ratio of 29:1, as reported by Jiang and coworkers^49^ (see the method section for further details). Then we performed creep experiments to quantify the Young's modulus and the power law exponent (*β*) of both neuronal microcompartments, which were compared to rheological parameters obtained on stiff PDMS substrates (E ~ 500 kPa). Our results did not show statistical significant differences in the rheological behaviour of bipolar cortical neurons grown on stiff PA and stiff PDMS substrates (Supplementary Fig. S7). Taken together, these results suggest that the different chemical natures between PA and PDMS matrices did not affect the mechanical properties of neuronal microcompartments. As a consequence, the PA hydrogel can be considered as a valuable material to change the matrix rigidity.

**Figure 5:**
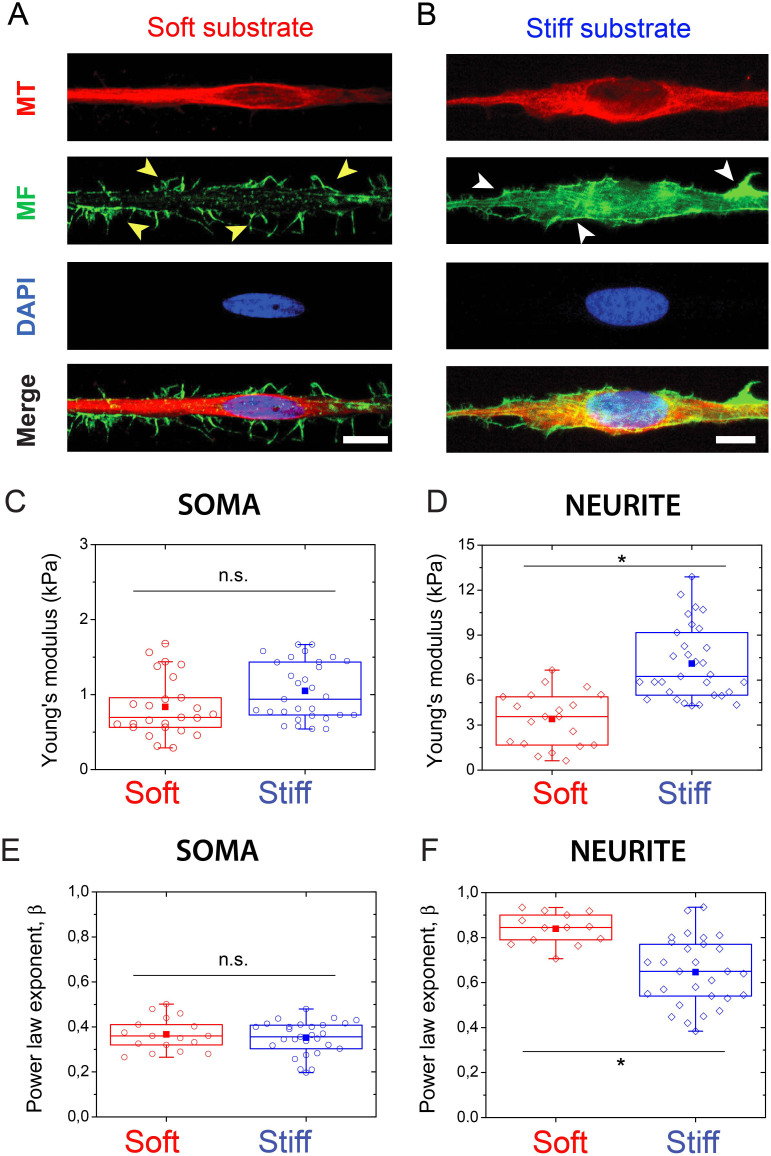
Mechanical adaptation of neuronal microcompartments to matrix stiffness changes. Immunostained cortical neurons plated on (A) soft (*E* = 3.5 kPa, left panel) and (B) stiff (*E* = 500 kPa, right panel) substrates show significant differences in the structure and organization the actin network (yellow and white arrows). F-actin is stained in green with Alexa-fluor 488, microtubules are stained in red with Alexa-fluor 561 and the nucleus is stained in blue with DAPI. Evolution of the Young's modulus of (C) the soma and (D) the neurite compartments of cortical neurons grown on soft (*E* = 3.5 kPa, in red) and stiff (*E* = 500 kPa, in blue) matrices. Evolution of the power law exponent *β* of (E) the soma and (F) the neurite compartments of cortical neurons grown on soft (*E* = 3.5 kPa, in red) and stiff (*E* = 500 kPa, in blue) matrices. Asterisks indicate significant changes (p < 0.01). The scale bars correspond to 10 μm.

We then decreased the Young's modulus of PA hydrogels to obtain soft microprinted culture substrates of 3.5 kPa. Previous studies have shown that non-neuronal cell types grown on stiff substrates were characterized by larger spreading areas and denser stress fibers^50^,^51^,^52^. For cortical neurons, we observed that numerous filopodium-like protrusions grown perpendicular to the long cell axis on soft substrates (Fig. 5A, yellow arrows), whereas stiff substrates were characterized by an actin-rich lamellipodia (Fig. 5B, white arrows). Interestingly, our results showed that the Young's modulus of the soma compartment of neurons grown on soft substrates was not statistically different than for those cultured on stiff substrates (Fig. 5C). This observation confirms our mechanical picture of the soma microcompartment, which is mainly controlled by the mechanical properties of the nucleus. In contrast, we observed a significant mechanical response of the neurite microcompartment to substrate stiffness, where neurites grown on soft hydrogels were two-times softer (Fig. 5D). Our findings show that the neurite microcompartment tuned its internal stiffness to match the compliance of the substrate, as observed previously for fibroblasts^53^. According to our previous investigations, we can suggest that this mechanoadaptive process requires the reorganization of the network of MTs, which control the elastic behavior of the neurite compartment.

We further assessed whether the viscoelastic state of neuronal microcompartments depended on matrix stiffness. We found that the *β* exponent for the soma was statistically not affected by matrix stiffness changes (Fig. 5E), confirming that the soma is not a mechanosensitive compartment of cortical neurons. In contrast, the power law exponent of neurites increased significantly (*β* = 0.85 ± 0.07) on soft substrates (Fig. 5F), suggesting that neurites become more fluid-like in soft microenvironments.

## Discussion

Our findings demonstrate that cortical neurons are composed of two microcompartments with opposite mechanical behaviors. This mechanical contrast leads to dissipation of a large amount of the energy propagated by a mechanical injury in the neurite microcompartment through a fluidization process, whereas the soma stores the energy as an elastic solid. Interestingly, our results indicate that the dissipation of energy in the neurite rises disproportionately with the amount of applied stress, suggesting that axonal damage increases as a function of the force with a non-proportional dependence. Our results report a body of evidence suggesting that the preferential damage of the neurite microcompartment observed after a traumatic event is intricately linked to a remodeling of cytoskeletal filaments, especially NFs that are responsible for the neurite fluidization. As a consequence, maintaining the NF network integrity could be an interesting target for TBI prophylactic drugs.

In the cell body, local deformation induces a significant condensation of chromatin, which has been identified as a key feature of chromatolysis leading to neuronal cell death after TBI^54^. It has been shown recently that the condensation of chromatin resulting from nuclear shape remodeling leads to a force-dependent stiffening of the nucleus^32^, providing a robust explanation of the stress stiffening behavior of the soma. Our mechanical picture suggests that cytoskeletal filaments and associated molecular motors play a minor role in the viscoelastic state of the soma, whereas the nucleus is the main determinant of the mechanical response of the cell body. Since it is well accepted that forces acting on the nucleus can be transduced in biological signals, our results suggest to pay more attention to pathological loading of neuron cell bodies that may arise in gray matter injuries.

In addition, our findings show that the cell body of cortical neurons remains soft and solid-like, regardless the matrix stiffness. This weak mechanical response of the cell body to matrix stiffness changes is in agreement with our mechanical picture of the cell body that emphasizes an important contribution of the nucleus^55^. Interestingly, Benzina and coworkers recently suggested that mechanical analysis of the soma differentiates neurons exhibiting regenerative growth from control neurons^56^. This work underlines the importance of preserving the neuronal cell body from pathological loads and supports our mechanical picture of the soma.

On the other hand, we demonstrated that cortical neurons possess a rigidity-sensing neurite compartment that becomes softer and adopts a pronounced viscous state on soft microenvironments. Our findings confirm recent results obtained by Atomic Force Microscopy (AFM) that report different stiffnesses between the cell body and neurites^57^. In addition, our findings help to interpret these observations by identifying the cytoskeletal components that are responsible for the mechanical properties in both neuronal microcompartments.

The significant fluidization of the neurite microcompartment on soft substrates reflects a cytoskeletal adaptation to substrate stiffness, which supports the fact that neurite outgrowth and viability of cortical neurons are intimately linked to the mechanical properties of the cellular surrounding^58^. Opposite rheological and mechanosensitive properties of the neuron microcompartments must be taken into account for understanding the onset of TBI events and identifying neurite outgrowth mechanisms. Furthermore, future studies must also consider the role of extracellular stiffness, which has been recently identified as a physical factor that determines cell fate in the cerebral cortex^59^. In this context, it is also important to underline the role of glial cells that contribute to a compliant environment for neurons^60^. Indeed, the biomechanical properties of glial cells may play a central role during brain injury by protecting neurons and then facilitating their outgrowth.

Collectively, our results highlight the importance of the regionalization of mechanical and rigidity sensing properties within neuronal microcompartments. Indeed, opposite rheological properties of neuronal microcompartments potentiate the higher vulnerability of neurites to mechanical injury and the ability of the cell body to preserve the integrity of genetic informations stored in the nucleus. Further investigations on the effect of mechanical injuries directed to specific neuronal microcompartments on electrophysiological activity will yield unique insights into functional deficits associated with TBI.

## Methods

### Cell culture

Primary rat cortical neurons (RCN) that come from the cortex of day-18 rat embryos (Life Technologies, Gaithersburg, MD) were suspended in a culture medium (Neurobasal Medium, Life Science) supplemented with B-27, 1% antibiotic/antimycotic and 5 mL GlutaMAX-I. Cells were seeded on LM microprinted substrates at a density of 10,000 cells per cm^2^. Samples were incubated under standard conditions at 37°C and 5% CO_2_. Media was replaced every 48 hours until experiments were executed. All experiments were performed on either day 4 or 5 post seeding.

### Culture substrates

RCN were cultured on stiff polydimethylsiloxane (PDMS) with a Young's modulus of 500 kPa and soft polyacrylamide hydrogels (hydroxy-PAAm)^46^ with a Young's modulus of 3.5 kPa. Both substrates were coated with laminin (Tebu-bio, Le Perray en Yvelines, France) stripes of 10 μm width by using a microcontact printing technique. These LM lines create highly ordered cell matrices for guidance of neurons that impose the direction of neurite extension and leads to the development of a bipolar neuronal morphology. Uncoated regions were blocked by incubating PDMS substrates for 5 min in a 1% Pluronic F-127 solution (BASF, Mount Olive, NJ) and hydroxy-PAAm hydrogels in a Bovine Serum Albumine (BSA) solution (5 mg/mL in PBS) during one night at 4°C while gently rocked. After blocking, both substrates were washed three times with a sterile PBS solution. Very stiff hydroxy-PAAm hydrogels were prepared as reported previoulsy^49^ for PA gels. Briefly, we increased the polyacrylamide monomer concentration to 30% with a fixed monomer/crosslink ratio of 29:1, leading to the reticulation of very stiff hydroxy-PAAm hydrogels of ~425 kPa.

### Magnetic tweezers

The magnetic tweezer was fabricated using a permalloy core (Permenorm 5000 H2 alloy, Vacuumschmelze GmbH, Hanau, Germany) that was equipped with a home-made 1000-turn solenoid. The tweezer was mounted on an inverted Nikon Eclipse Ti microscope (Nikon, Japan) and was controlled by an automatized micromanipulator system (InjectMan NI2, Eppendorf, Hamburg, Germany). Current in the solenoid was produced by a voltage-controlled current supply (Kepco Model BOP 20–20 M, Flushing, NY) that transformed voltage signals from a function generator into a current signal with amplitudes up to 5A. The power supply was connected to the terminals of the solenoid and a LabVIEW software (National Instruments, Austin, Texas) was used to generate the desired voltage waveform. The magnetic tweezer was positioned at a 25-degree angle to the microprinted culture substrate by using an automated micromanipulator system (InjectMan NI2, Eppendorf, Hamburg, Germany). This position permits the application of magnetic forces with shallow angle in order to minimize out of plane bead displacements. As the beads remained in the focal plane during creep experiments, we could confirm that bead displacements were confined to the *xy* plane. The magnetic tweezer was calibrated by recording the displacement of paramagnetic beads (4.5 μm in diameter) in a 99% glycerol solution for various current levels (Supplementary Movie S5). Utilizing Stoke's formula and magnetic bead velocities, we calculated force as a function of distance from tweezer tip for each current level (Supplementary Fig. S8). Force calibration curves were used to maintain the bead to tip distance between 60 μm and 120 μm to ensure a linear regime of forces, depending on the current level. The range of bead to tip distances was not higher than 120 μm to have a magnitude of force that depended only on the Euclidean distance to the tip, and is independent of the bead position relative to the needle axis^15^. In addition, we fixed a minimal bead to tip distance of 50 μm to minimize the effect of the nonlinearity of the force-distance relationship and also to prevent out of plane (*x,z*) bead displacements. Taken together, these experimental conditions ensure to preserve in-plane (*x,y*) bead displacements and a constant applied force value. Temperature rise of the extracellular media as a result of the Joule effect in the tweezer's coil was determined to be approximately one degree Celsius.

### Bead functionalization

We used BcMagTM Epoxy-Activated Magnetic beads (Bioclone Inc., San Diego, CA) of 4.5 μm in diameter that were pre-activated, uniform and silica-based beads coated with high-density epoxy functional groups on the surface. Epoxy-activated beads bind nucleophiles such as hydroxyl groups within proteins, providing an extremely stable linkage between the ligand and the matrix. In order to bind superparamagnetic beads specifically to the cytoskeleton of neurons, the bead surface was coated with human fibronectin (FN) by following the bead manufacturer's protocol for bead functionalization. Beads were first rinsed in phosphate buffered saline (PBS) and subsequently incubated on ice in 10 μg/mL human FN overnight under a gentle (0.5 Hz) agitation to disperse beads without precipitating FN still in solution. After rinsing in PBS, FN-coated beads were seeded onto neurons in culture medium for 20 minutes at a mean ratio of 4 beads per neuron.

### Pharmacological agents

Specific pharmacological agents were first used to inhibit the polymerization of microtubules^25^ (Colchicine, Enzo Life Sciences, Zandhoven, Belgium), to disorganize neurofilaments^26^ (ß-ß'-iminodipropionitrile, IDPN, Sigma Aldrich, St Louis, MO) and actin filaments^27^ (latrunculin-A, Enzo Life Sciences, Zandhoven, Belgium). Colchicine was applied at 1 μM for 20 min, IDPN at 5% w/v for 20 min. and latrunculin at 10 μM for 30 min. In addition, we used Blebbistatin (Enzo Life Sciences, Zandhoven, Belgium) at 75 μM during 20 min. to inhibit myosin II contractility^61^, monastrol (Sigma Aldrich, St Louis, MO) at 100 μM during 30 min. to inhibit the ATPase activity of kinesin 5^62^, erythro-9-(2-hydroxy-3-nonyl)adenine (EHNA, Sigma Aldrich, St Louis, MO) at 100 μM during 20 min. to inhibit the ATPase activity of the cytoplasmic dynein^63^ and 2,5-hexanedione (Sigma Aldrich, St Louis, MO) at 10 mM during 30 min. to increase the cross-linking of neurofilaments^64^. Control experiments were performed with DMSO up to 0.5% during 30 min. and additional experiments demonstrated that the effects of all of these pharmacological agents were sustained for the entire experimental period.

### Immunofluorescent staining

Immunostained preparations were observed in epifluorescence and confocal mode with a Nikon Eclipse Ti-E motorized inverted microscope (Nikon, Japan) equipped with ×40 Plan Apo (NA 1.45, oil immersion), ×60 Plan Apo (NA 1.45, oil immersion) and ×100 Plan Apo (NA 1.45, oil immersion) objectives, two lasers (Ar-ion 488 nm; HeNe, 543 nm) and a modulable diode (408 nm). Epifluorescence images were captured with a Roper QuantEM:512SC EMCCD camera (Photometrics, Tucson, AZ) using NIS Elements AR (Nikon, Japan) and confocal images were acquired with a Nikon C1 scanhead by using small Z-depth increments between focal sections (0.15 μm).

### Statistical analysis

The calculated value for each group of variables is presented in terms of mean value and standard deviation (Mean ± SD). To test pairwise differences in population experiments, Student's t-tests were performed between individual treatments with OriginPro 8.5. Values of *p* < 0.01 compared to control were considered statistically significant and are indicated by asterisks in the graphs. N.S. indicates no statistical significance.

## Author Contributions

T.G., B.E.D., K.K.P. and S.G. designed the study. T.G. and B.E.D. performed experiments. T.G. and S.G. analyzed data and wrote the manuscript. B.E.D. and K.K.P. improved the manuscript and the figure presentation. All authors read, commented and approved the manuscript.

## Supplementary Material

Supplementary Information Supplementary Information

Supplementary Information Movie S1

Supplementary Information Movie S2

Supplementary Information Movie S3

Supplementary Information Movie S4

Supplementary Information Movie S5

Supplementary Information Movie S6

Supplementary Information Movie S7
