# Supplementary material for: Opposite rheological properties of neuronal microcompartments predict axonal vulnerability in brain injury
Source: Sci Rep. 2015 Mar 30;5:9475. doi: 10.1038/srep09475 (PMC4377573; doi:10.1038/srep09475)
Supplement: Supplementary Information [file srep09475-s1.pdf]

Supporting Information

Opposite rheological properties of neuronal microcompartments predict axonal vulnerability in brain injury

Thomas Grevesse[1,2], Borna E. Dabiri[2], Kevin K. Parker[2] and Sylvain Gabriele[1*]

[1] Mechanobiology & Soft Matter group, Laboratoire Interfaces et Fluides Complexes, Centre d'Innovation et de Recherche en Matériaux Polymères (CIRMAP), Research Institute for Biosciences, Université de Mons, 20, Place du Parc, B-7000 Mons, Belgium.

[2] Disease Biophysics Group, Wyss Institute for Biologically Inspired Engineering, School of Engineering and Applied Sciences, Harvard University, 29 Oxford Street, Cambridge, MA 02138, USA.

[*]To whom correspondence should be addressed. E-mail: sylvain.gabriele@umons.ac.be

Expression of the creep function

The creep function *J(t)* is defined as the ratio between the strain *ε(t)* and the stress σ_0, as:

$$J(t) = \frac{\varepsilon(t)}{\sigma_0} \quad (1)$$

Taking the specific morphology of neurons into account, *ε(t)* and σ_0 must be defined for both microcompartments accordingly to their specific geometries (Fig. S1):

• **The soma:** the strain, *ε(t)*, of the soma corresponds to the bead displacement, *d(t)*, normalized by the short axis of the soma, r_{soma}. The stress, σ_0, corresponds to the constant force applied on the paramagnetic bead, f_0, divided by the surface of force application which is the bead cross-section, such as:

$$J_{soma}(t) = \frac{d(t)}{r_{soma}} \frac{\pi r_{bead}^2}{f_0} \quad (2)$$

Assuming a Poisson's ratio $\nu = 0.5$ for mammalian cells, the Young's modulus of the soma, E_{soma}, can be estimated from the shear modulus $G=1/J_{soma}$, as:

$$E_{soma} = 2G(1+\nu) = \frac{2}{J_{soma}}(1+\nu) \quad (3)$$

• **The neurite:** the strain, *ε(t)*, corresponds to the elongation of the neurite segment $\Delta(l)=l(t)-l_0$ normalized by its initial length, l_0. The stress, σ_0, corresponds to the constant force applied on the paramagnetic bead, f_0, divided by the neurite cross-section, such as:

$$J_{neurite}(t) = \frac{l(t) - l_0}{l_0} \frac{\pi r_{neurite}^2}{f_0} \quad (4)$$

Where the deformed neurite length, *l(t)*, is derived from the bead displacement, *d(t)*, and the initial neurite length, l_0, such as:

$$l(t) = \sqrt{4d^2(t) + l_0^2} \quad (5)$$

Considering the geometry of the neurite deflection, the inverse of the prefactor, $1/J_0$, is equivalent to a Young's modulus.

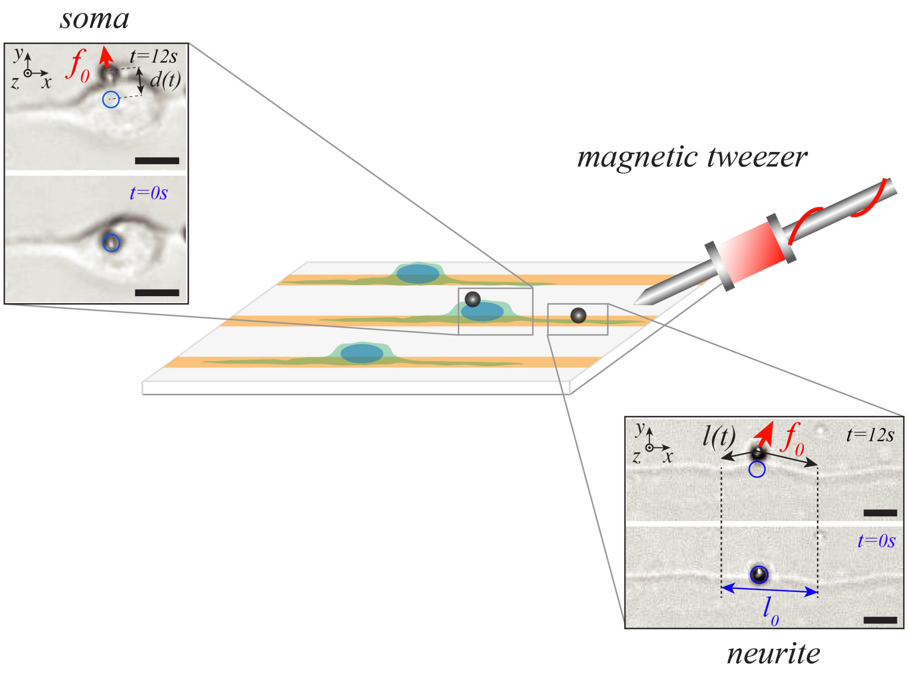

Figure S1: Schematic representation of the local deformations of the soma and the neurite microcompartments during a creep experiment performed with magnetic tweezers on bipolar cortical neurons. Phase-contrast images show the displacement of a FN-coated paramagnetic bead bound to the soma (left box) and the neurite (right box). Scale bars correspond to 10 μm.

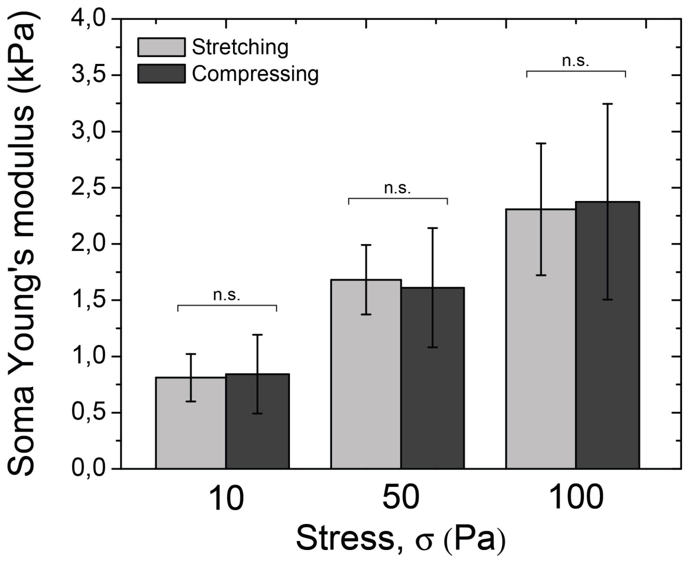

Figure S2: Evolution of the Young's modulus of the soma microcompartment as a function of the local stress applied in stretching (light gray columns) or compressing (dark gray columns) mode. Stress-stiffening of the soma can be observed in both deformation modes. N.S. indicates no statistical significance.

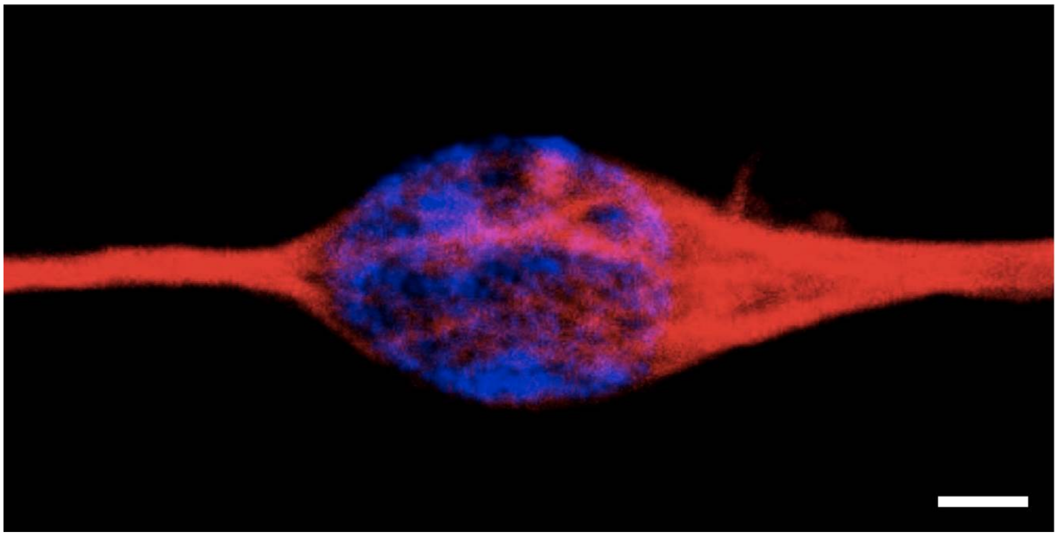

Figure S3: Confocal image of the spatial organization of the microtubule network in the cell body microcompartment of a bipolar cortical neuron. Microtubules (stained in red with rhodamine) are wrapped around the nucleus (stained in blue with DAPI). The scale bar corresponds to 10 μm.

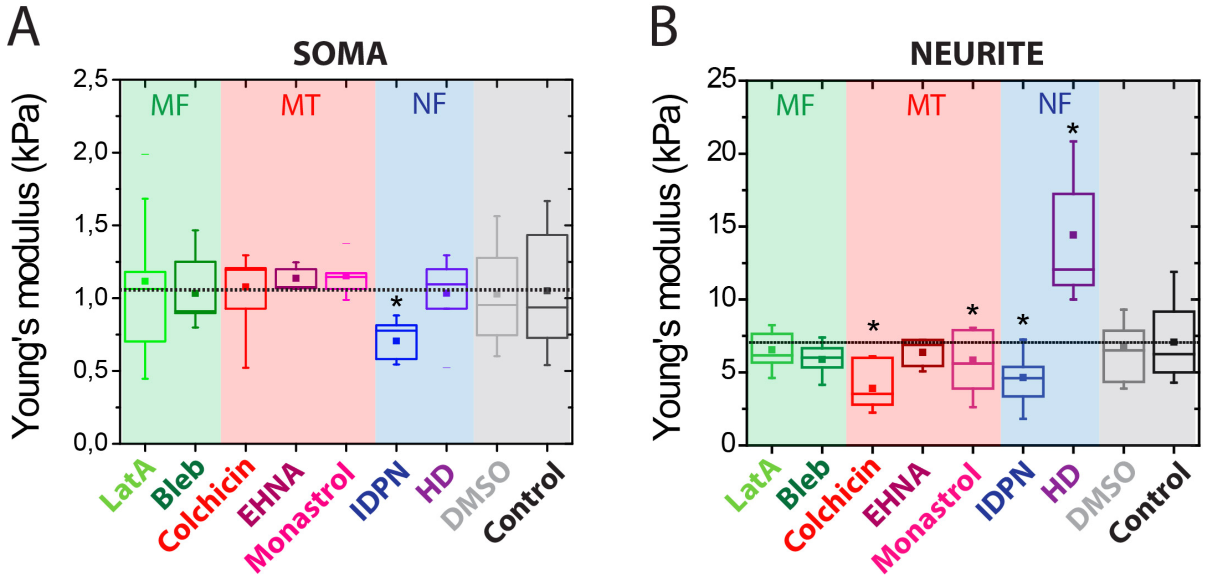

Figure S4: Evolution of the Young's modulus of (A) the soma and (B) the neurite microcompartments in response to the selective disruption of cytoskeletal components and inhibition of molecular motors ($12 \leq n \leq 14$). The black dashed lines correspond to the mean Young's modulus value of (A) the soma and (B) the neurite microcompartments of control cells ($n = 31$). Asterisks indicate significant changes ($p < 0.05$).

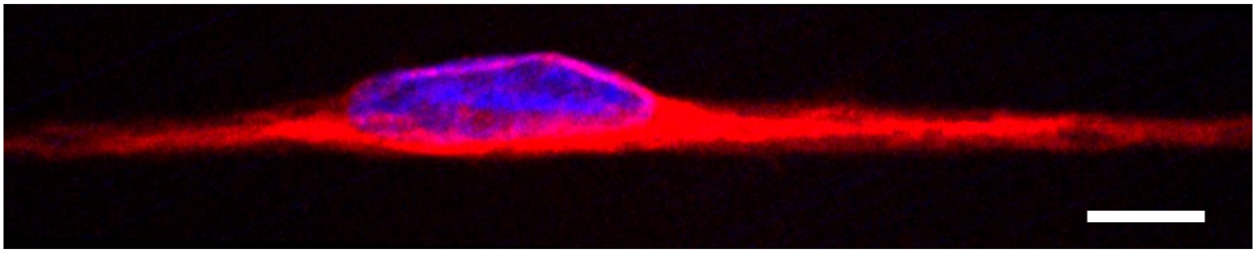

Figure S5: Image of a bipolar cortical neuron plated on soft 3.5 kPa hydroxy-PAAm substrates microcontact printed with 10 μm LM lines. Microtubules are stained in red with rhodamine and the nucleus is stained in blue with DAPI. The scale bar corresponds to 5 μm.

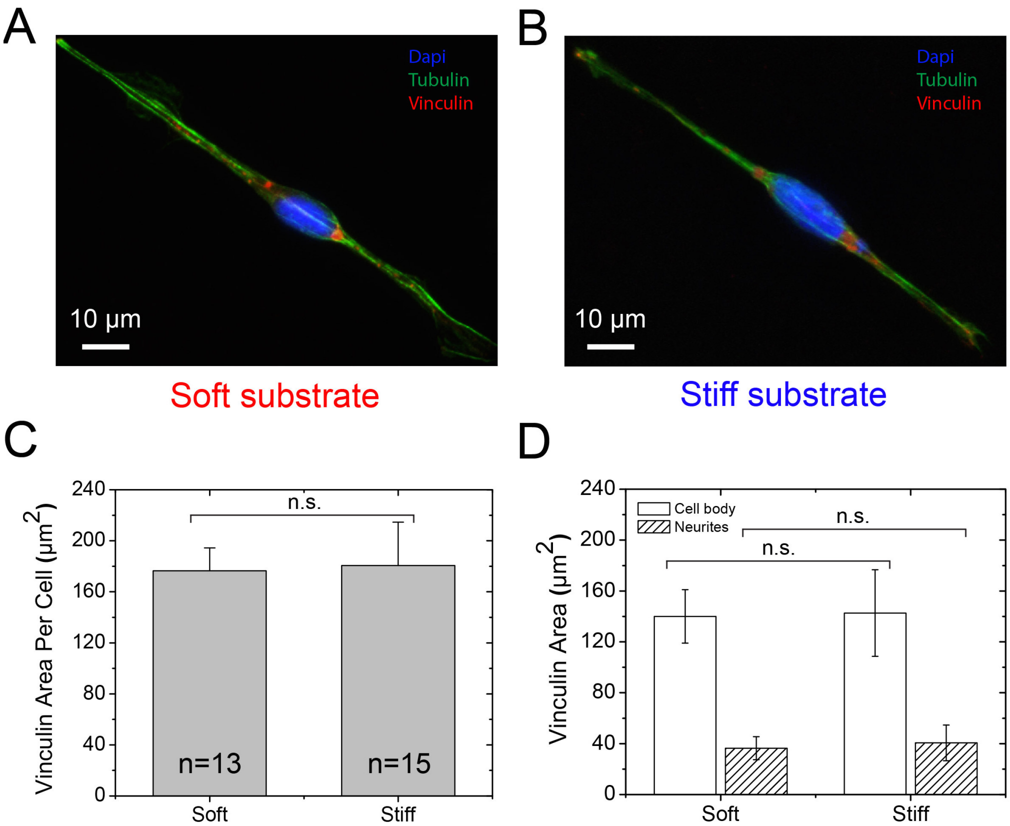

Figure S6: Immunostaining image of a bipolar neuron grown on a 10 μm wide LM line deposited on (A) a soft hydroxy-PAAm hydrogel (E=3.5 kPa) and (B) a stiff PDMS substrates (E=500 kPa). Neurons are stained for DAPI in blue, tubulin in green and vinculin in red. Scale bars are 10 μm. (C) Quantification of the total vinculin area per cell for bipolar neurons on soft (n=13) and stiff (n=15) culture substrates. (D) Repartition of the vinculin area for the cell body (plain bars) and the neurite (dashed bars) microcompartment on soft and stiff matrices. N.S. indicates no statistical significance.

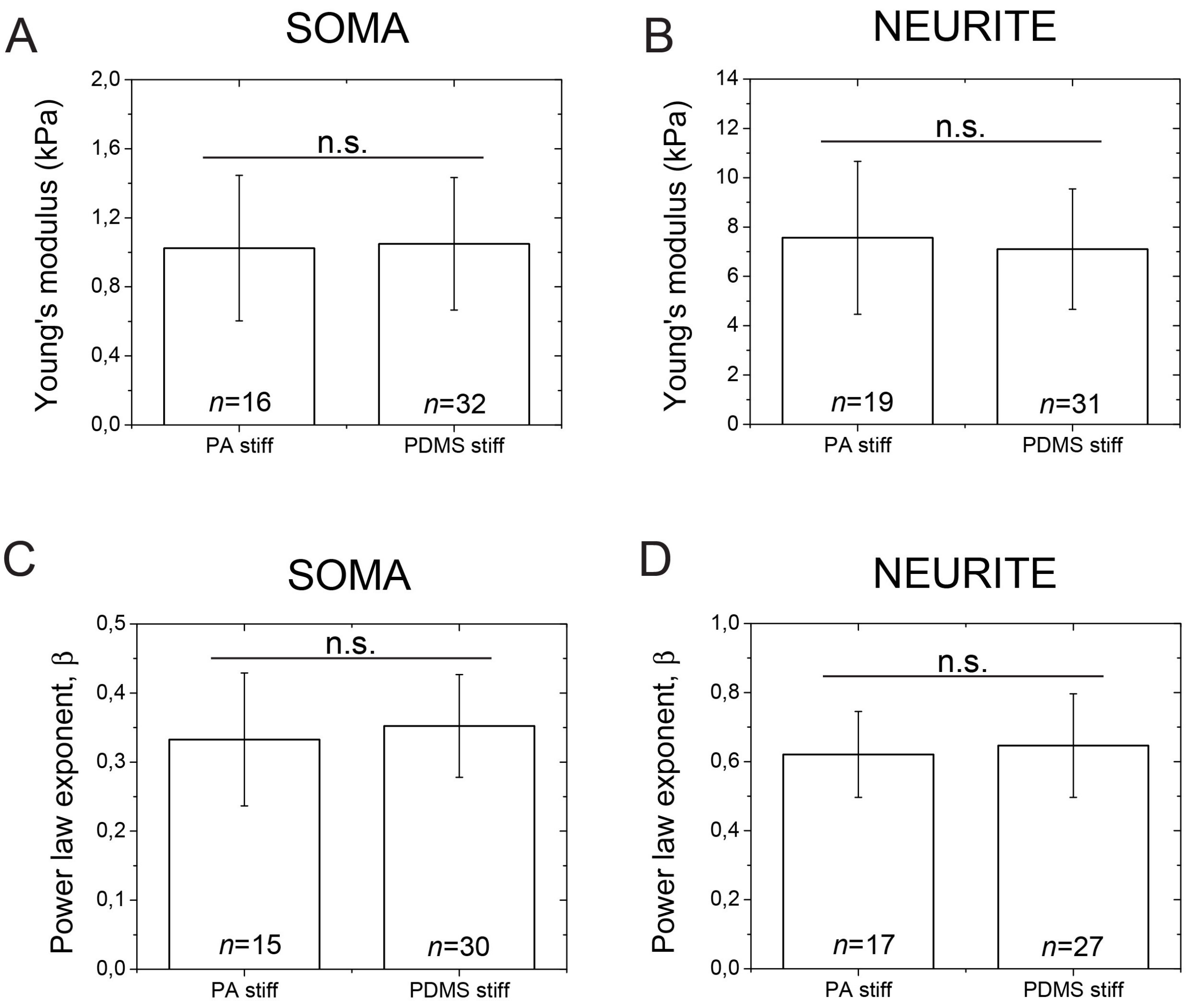

Figure S7: Rheological characterization of the soma (A and C) and the neurite (B and D) microcompartments of bipolar neurons grown on 10 μm LM lines deposited on stiff hydroxy-PAAm hydrogels (E=425 kPa) and stiff PDMS substrates (E=500 kPa). N.S. indicates no statistical significance.

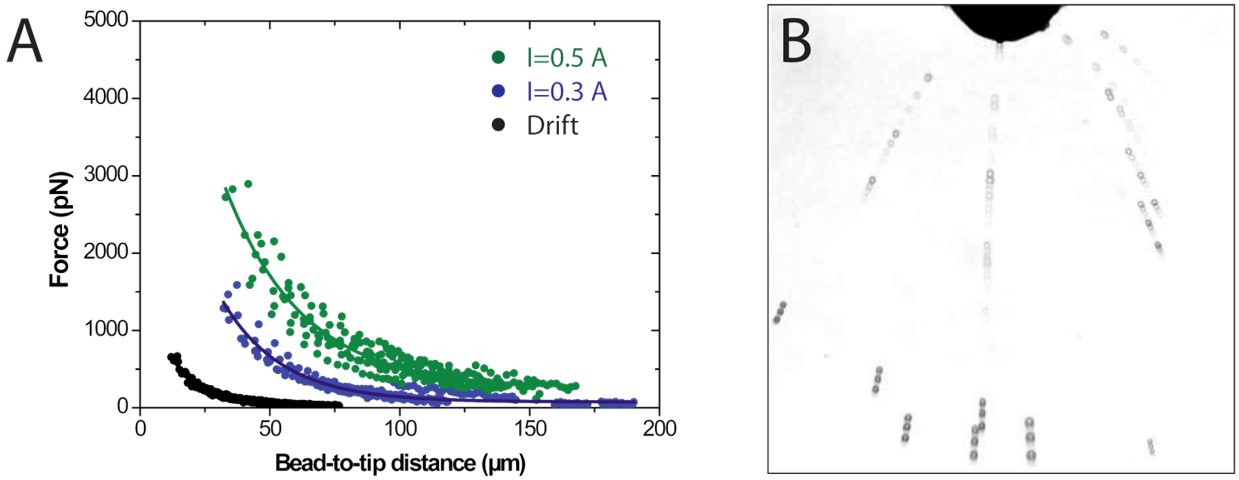

Figure S8: (A) The force calibration curve of the home-made magnetic tweezer set-up was obtained by tracking (B) the velocity of 4.5 μm paramagnetic beads in a 99% glycerol solution, based on the Stokes formula for low Reynolds number flow.

Movie S1: Representative displacement of a FN-coated paramagnetic bead bound to the soma micro-compartment of a bipolar cortical neuron in response to a constant pulling force imposed by magnetic tweezers. The scale bar corresponds to 10 μm.

Movie S2: Representative displacement of a FN-coated paramagnetic bead bound to the neurite micro-compartment of a bipolar cortical neuron in response to a constant pulling force imposed by magnetic tweezers. The scale bar corresponds to 10 μm.

Movie S3: Serial confocal micrographs (Z-axis scanned) for the soma micro-compartment of a cortical neuron stained for DNA (in blue) and microtubules (in red). Cross-sections are collected from the top to the underneath of the cell.

Movie S4: Evolution of the fluorescence intensity of the Hoechst staining within a nucleus of a cortical neuron in response to a typical creeping experiment performed on the soma micro-compartment.

Movie S5: Serial confocal micrographs (Z-axis scanned) of a cortical neuron plated on 3.5 kPa hydroxy-PAAm substrate and stained for DNA (in blue), microtubules (in red) and actin filaments (in green). Cross-sections are collected from the top to the underneath of the cell. Z-step corresponds to 0.2 μm.

Movie S6: Serial confocal micrographs (Z-axis scanned) of a cortical neuron plated on 500 kPa PDMS substrate and stained for DNA (in blue), microtubules (in red) and actin filaments (in green). Cross-sections are collected from the top to the underneath of the cell. Z-step corresponds to 0.4 μm.

Movie S7: The magnetic tweezer was calibrated by recording the displacement of paramagnetic beads (4.5 μm in diameter) in 99% glycerol toward the tip of the tweezer.
